# Reliability and reproducibility of the venous excess ultrasound (VExUS) score, a multi-site prospective study: validating a novel ultrasound technique for comprehensive assessment of venous congestion

**DOI:** 10.1186/s13054-024-04961-9

**Published:** 2024-06-11

**Authors:** August A. Longino, Katharine C. Martin, Katarina R. Leyba, Luke McCormack, Gabriel Siegel, Vibhu M. Sharma, Matthew Riscinti, Carolina O. Lopez, Ivor S. Douglas, Edward A. Gill

**Affiliations:** 1 Department of Internal Medicine, University of Colorado Hospital, 12631 E 17th Avenue, Aurora, CO 80045 USA; 2 Department of Emergency Medicine, University of Colorado Hospital, Aurora, CO USA; 3 Department of Pulmonary and Critical Care Medicine, University of Colorado Hospital, Aurora, CO USA; 4 Department Hospital Medicine, University of Colorado Hospital, Aurora, CO USA; 5 Department of Pulmonary and Critical Care Medicine, Denver Health Medical Center, Aurora, CO USA; 6 Department of Cardiology, University of Colorado Hospital, Aurora, CO USA

## Abstract

Though the novel venous excess ultrasound (VExUS) score is increasingly used as a noninvasive means of venous congestion measurement, the inter-rater reliability (IRR), inter-user reproducibility (IUR), and utility of concurrent ECG have not been evaluated. We conducted a multicenter study of the IRR, IUR, and utility of ECG for VExUS interpretation between four attending physicians of diverse specialties, reporting the Kappa statistic (KS) and Intraclass Correlation Coefficient (ICC) for IRR and IUR for scans with and without ECG. Eighty-four paired VExUS exams from 42 patients, 60 of which had a concurrent ECG tracing, were interpreted. They showed substantial IRR, with a KS of 0.71 and ICC of 0.83 for the overall VExUS grade (*p* < 0.001), and IUR, with a KS 0.63 and ICC of 0.8. There was greater agreement among images with an ECG tracing. These results suggest that ECG-augmented VExUS may be a reliable and reproducible measure interpretable by clinicians with diverse backgrounds.

## Background

Historically, the medical community has focused on the arterial side of the circulation. However, pathologic venous congestion is increasingly recognized an under-appreciated cause of harm in multiple patient populations [1–3]. Despite the importance of this clinical parameter, assessment of venous congestion remains challenging, as conventional physical exam findings depend on patient characteristics and provider experience. While ultrasound of the inferior vena cava (IVC) was thought to address these issues, studies have shown lower-than expected clinical utility [4]. Recognizing these barriers, clinicians often rely on right heart catheterization (RHC), the gold standard for assessment of venous congestion. Unfortunately, RHC is invasive, resource-intensive, and unavailable in many centers [5]. Central venous pressure (CVP), another common proxy for venous congestion, is also unavailable in many patients. These limitations demonstrate the need for a reliable, cost-effective, noninvasive means of measuring venous congestion.

To that end, Beaubien-Souligny and colleagues developed the novel “venous excess ultrasound (VExUS)” Score. The authors described a noninvasive 4-point exam combining IVC measurement with Doppler ultrasonography of the hepatic vein (HV), portal vein (PV), and renal veins (RV), and reported a positive likelihood ratio of 6.37 for cardiorenal acute kidney injury (AKI) [2, 3]. Since that time multiple reviews have been published on the use of VExUS, with a focus on its clinical utility for assessment of venous congestion [6–8]. Subsequent validation studies found that VExUS correlates with AKI and invasively-measured intracardiac pressures [9, 10]. These findings have generated considerable interest in the technique, and including multiple prospective trials evaluating its utility [11, 12]. Despite its rapid adoption, much remains unknown about the VExUS score. An essential validation step for any ultrasonographic technique is assessment of inter-rater reliability (IRR), consistency of image interpretation between interpreters, and inter-user reproducibility (IUR), consistency of result interpretation when data from one patient is collected by multiple ultrasonographers. In the case of VExUS, there has also been a question of whether a concurrent electrocardiogram (ECG) tracing improves interpretability. Best practices and standardized protocols have not been established, and real-world implementation of VExUS varies widely. To address these gaps in the literature, we conducted a multi-center, multidisciplinary prospective observational study to assess the IRR, IUR, and necessity of ECG for VExUS interpretation.

## Methods

A convenience sample of patients from two tertiary medical centers were enrolled. Inclusion criteria were inpatient admission and ability to tolerate a VExUS exam. Exclusion criteria included pregnancy or inability to provide informed consent. Inclusion criteria were kept deliberately broad to enroll patients with a variety of pathologies and VExUS grades in order to assess the IRR and IUR of VExUS in a broad population. Patients with conditions that might confound VExUS (e.g., cirrhosis) were included to increase generalizability of findings. For assessment of IUR, two sets of VExUS images were acquired per patient by different ultrasonographers blinded to each other’s exams. Ultrasonographer order was randomized. Ultrasonographers were 4 internal medicine residents that underwent a 4-h online training course in VExUS, followed by 3 in-person practice sessions from an attending physician with board-certification in ultrasound and specific focus on VExUS. Anonymized VExUS images were then interpreted by a four-member interpretation team including a pulmonologist/intensivist, cardiologist, emergency medicine physician, and hospitalist, each board-certified in their specialty of origin. All members of the interpretation team had demonstrated competence with the VExUS technique, having undergone the same training as the ultrasonographers and performed 20–100 VExUS exams. Interpreters provided a grade for each exam component, an overall score. For purposes of quality control, interpreters gave each set of images an assessment of image quality, evaluated on a 1–5 scale recommended by the American College of Emergency Physicians [13]. An ECG tracing was added to the protocol for the last 30 patients enrolled. Exams took place within a 20-min window to avoid changes in venous congestion.

### VExUS protocol

Following acquisition of informed consent, VExUS exams were conducted as previously described [2]. Ultrasonographers measured IVC diameter 1–2 cm caudal to the confluence of the HV and IVC. HV pulsatility was assessed in a subxiphoid or lateral view, placing the Doppler gate across any of the hepatic veins. Portal venous pulsatility was assessed by placing the Doppler gate across the PV and observing waveform pulsatility index. Renal vasculature was visualized with the probe in the posterior axillary line, with the Doppler gate placed to detect flow in the interlobar vessels in the renal cortex. Exams were conducted using the abdominal probe of the Mindray TE7 (Mindray Bio‐Medical Electronics Co).

### VExUS scoring

The VExUS score is composed of evaluations of the IVC, HV, PV, and RV [2]. If a patient’s IVC diameter is < 2 cm, the exam is assigned a score of 0, (no congestion). In the presence of an IVC ≥ 2 cm, the examiner categorizes each vein as either normal, mildly abnormal, or severely abnormal. all views were acquired in all patients. Normal HV Doppler waveforms show a small, retrograde a-wave, followed by anterograde systolic (S) and diastolic (D) waves, with the ratio of the S:D waves being > 1. In increasing states of congestion, the S wave shrinks relative to the D wave before reversing entirely, becoming retrograde. A S:D ratio > 1 is normal, a S:D ratio ≤ 1 is mildly abnormal, and S wave reversal is severely abnormal. A normal PV Doppler waveform shows pulsatility of < 30%. Pulsatility of 30–49% is mildly abnormal, and a pulsatility > 50% is severely abnormal. A normal RV Doppler pattern shows continuous, non-pulsatile anterograde flow. A continuous venous baseline is normal. Biphasic pulsations during systole and diastole are considered mildly abnormal. Monophasic pulsation during diastole is severely abnormal.

Any combination of normal or mildly abnormal scores is given a grade of 1. If the patient has one severely abnormal score, they are given a grade of two. Two or more severely abnormal scores results in a grade of 3, reflecting severe congestion.

### Data analysis

Exams with an image quality score of > 2 were included. We calculated Light’s Kappa Statistic (KS) and the two-way intraclass correlation coefficients (ICC) [14] to assess IRR between all four interpreters for grades of the overall VExUS exam and grades for each exam component. We then assessed IUR by comparing concordance between the first and second scans on each patient as reported by each interpreter and calculated mean and standard deviation of the ICC and KS of all 4 interpreters. Scores were presented as mean, standard deviation (SD), and highest p-value.

## Results

56 patients were enrolled in the study. Of these, 42 patients had quality scores > 2 for both VExUS exams and were included in the final analysis, allowing for comparison of 84 paired scans, a feasibility rate of 75%. Sixty scans had concomitant ECG tracings. Demographic and concordance statistics are presented in Tables 1 and 2. All patients were admitted to the general medicine wards. None were using positive pressure ventilation at the time of their examinations. Clinical characteristics that could confound the VExUS score (e.g., cirrhosis, abdominal tumors, tricuspid regurgitation) are listed in Table 1. Central Venous pressure monitoring was unavailable for the cohort.

**Table 1:** Cohort demographics

Characteristic	N = 42
Sex	
Male	29 (69%)
Female	13 (31%)
Age (years)	64 (54, 69)
BMI	30 (25, 40)
History of heart failure with reduced ejection fraction	23 (55%)
Most recently documented ejection fraction (%)	35 (20, 45)
History of heart failure with preserved ejection fraction	14 (33%)
History of tricuspid regurgitation	
Yes	18 (43%)
No	23 (55%)
Unknown	1 (2.4%)
Tricuspid regurgitation severity	
Mild	8 (47%)
Moderate	4 (24%)
Severe	5 (29%)
History of cirrhosis	5 (12%)
Ascites present on exam	4 (9.5%)
COPD	17 (40%)
Asthma	7 (17%)
Abdominal tumor	1 (2.4%)
History of end stage renal disease	2 (4.8%)
Charlson comorbidity index	5.00 (3.00, 6.00)
Length of stay (days)	5.0 (4.0, 10.3)
Reason for admission	
ACS	3 (7.1%)
Acute hypoxic respiratory failure	4 (9.5%)
Alcoholic Hepatitis	0 (0%)
Arrhythmia	0 (0%)
Decompensated cirrhosis	1 (2.4%)
Heart failure exacerbation	12 (29%)
Hyperglycemia	1 (2.4%)
Hypervolemia	14 (33%)
Malignancy	2 (4.8%)
Pulmonary embolism	0 (0%)
Scheduled cardiac catheterization	1 (2.4%)
Sepsis	2 (4.8%)
Small bowel obstruction	0 (0%)
Undifferentiated shock	2 (4.8%)

Continuous variables are presented as median (IQR); categorical variables are presented as n (%)

*BMI* Body mass index, *ACS* Acute coronary syndrome

**Table 2:** Concordance statistics

Concordance statistics					
Inter-rater reliability	Kappa statistic	*p*-value	ICC	CI	*p*-value
Total cohort: n = 84 VExUS scans					
VExUS grade	0.71	< 0.001	0.83	0.77–0.88	< 0.001
Hepatic vein	0.52	0.001	0.71	0.62–0.8	< 0.001
Portal vein	0.53	< 0.001	0.74	0.66–0.82	< 0.001
Renal vein	0.32	0.02	0.48	0.31–0.64	< 0.001
Without ECG tracing: n = 24 VExUS scans					
VExUS grade	0.423	0.006	0.59	0.26–0.88	< 0.001
Hepatic vein	0.18	0.49	0.26	− 0.1–0.83	0.108
Portal vein	0.47	0.47	0.69	0.36–0.93	< 0.001
Renal vein	0.31	0.27	0.39	− 0.019–0.88	0.04
With ECG Tracing: n = 60 VExUS scans					
VExUS grade	0.75	< 0.001	0.86	0.8–0.9	< 0.001
Hepatic vein	0.55	0.012	0.73	0.63–0.81	< 0.001
Portal vein	0.53	0.001	0.75	0.66–0.82	< 0.001
Renal vein	0.32	0.03	0.5	0.31–0.68	< 0.001

	KS	SD	ICC	SD	*p*-value
Inter-user reproducibility: n = 42 VExUS scan pairs					
VExUS grade	0.63	0.03	0.795	0.06689544	< 0.001
Hepatic vein	0.57	0.1	0.715	0.09394147	< 0.001
Portal vein	0.41	0.1	0.6125	0.09283722	< 0.001
Renal vein	0.38	0.07	0.5625	0.07258616	< 0.001

*VExUS* Venous excess ultrasound; *ECG* Electrocardiogram; *ICC* Intraclass correlation coefficient; *CI* Confidence Interval

### Inter-rater reliability

**Fig. 1:**
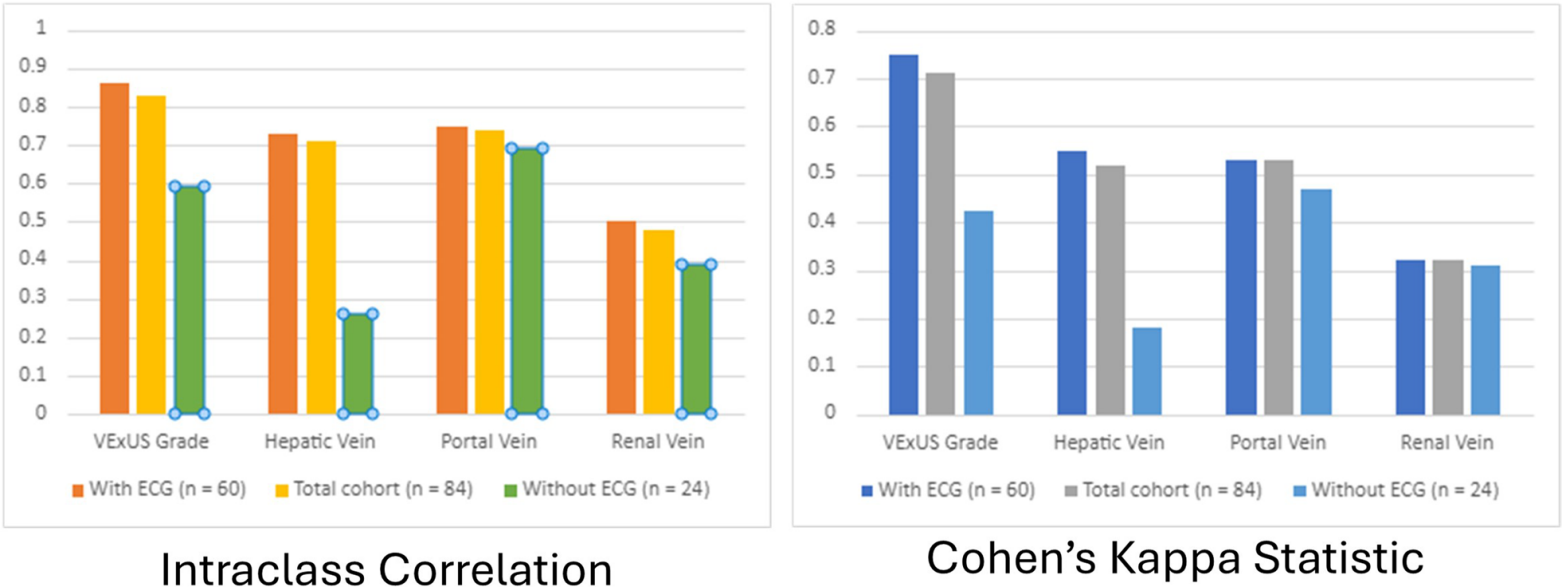
Inter-rater reliability and concordance of VExUS exam components.Concordance statistics for the cohort. Concordance as measured by intraclass correlation coefficient and Cohen’s kappa statistic was greater for the overall VExUS grade than each of its individual components. It was also greater among images with a concurrent electrocardiogram tracing than for images without one

The KS and ICC between interpreters was 0.71 and 0.83 for the overall VExUS grade (*p* < 0.001), suggesting substantial agreement [14]. Kappa statistics and ICCs for the individual VExUS components were lower than for the overall VExUS exam, ranging from fair to moderate; 0.52 and 0.71 for HV, 0.53 and 0.74 for PV, and 0.32 and 0.48 for RV (*p* < 0.03) (Fig. 1; Table 2). There was an increase in concordance with the ECG tracing, with a KS and ICC of 0.75 (*p* < 0.01) and 0.86 (*p* < 0.01) with an ECG lead compared to 0.42 (*p* < 0.01) and 0.59 (*p* < 0.01) without an ECG lead.

### Inter-user reproducibility

When comparing images from the same patient, the average ICC was 0.8 for VExUS grade, 0.72 for HV, 0.61 for PV, and 0.56 for RV. The average KS was 0.63 for VExUS grade, 0.57 for HV, 0.41 for PV, and 0.38 for RV (Table 2).

## Discussion

We found substantial IRR for the VExUS score between multiple readers and IUR between images acquired from the same patient by sequential ultrasonographers. The IRR of the VExUS exam as collected and interpreted by physicians was superior the reported IRR of IVC measurement in a group of emergency department physicians [15], and similar to the IRR of IVC measurements when collected by professional ultrasonographers [16]. Though substantial, the KS for the VExUS grade was lower than reported in a recent study of VExUS in patients with septic shock, which reported a KS of 0.95 (95% CI 0.90–1.0) between two experts [17]. This discrepancy may be attributable to differences in experience of interpreters, as well as the fact that the current study evaluated 4 interpreters instead of 2, and included a more heterogenous patient population. The overall VExUS exam demonstrated higher levels of IRR and IUR than individual components of the exam, suggesting that the redundancy of the VExUS score may compensate for variability of interpretation of individual components. The renal component of the exam had the lowest IRR and IUR, likely due to difficulty of acquiring and interpreting renal images, generally considered the most difficult to acquire. Further refinements of VExUS should robustly evaluate the performance characteristics of the renal view. In a recent study of IRR in renal and hepatic vasculature of healthy pregnant women this discrepancy was not observed [18], suggesting that the difference may be due to the patient population under study or operator or interpreter experience. The same study showed a marked increase in IRR with training, suggesting that additional instruction may be helpful for VExUS interpretation. While the current study included patients with a range of potentially-confounding comorbidities such as heart failure and portal hypertension, IRR and IUR will need to be rigorously observed in key subpopulations such as those with respiratory failure, positive pressure ventilation, and changes to intra-abdominal pressures.

The improved IRR following introduction of an ECG tracing for the final 30 patients is consistent with prior literature showing that ECG tracings increase IRR of venous doppler ultrasonography of the hepatic and renal vasculature,[18] possibly because the ECG tracing allows the interpreter to distinguish changes in waveforms due to the cardiac and respiratory cycles, a common source of confusion in VExUS interpretation. These results suggest that ECG improves VExUS readability, and should be considered as part of a standardized VExUS protocol. This may be a barrier to the wider implementation of VExUS, as ECG integration with non-echocardiographic bedside ultrasound is rare. The feasibility rate of 75% may be due to a variety of factors including patient body habitus and technique complexity. Though the VExUS technique has been used in a range of populations, it is possible that the IRR and IUR would decrease in a more medically complex population [2, 3, 6, 19], One factor that may impact IRR, IUR, and feasibility is the experience level of both scanners and interpreters- VExUS is a novel technique, and while few clinicians are currently familiar with its use, broader adoption and increased experience may lead to improvements in these important parameters.

Strengths of the current study include a diverse patient population from multiple centers, strengthening the generalizability of the findings to a broader population than VExUS is usually applied to, as well as a diverse interpretation team including multiple specialities, suggesting that VExUS is interpretable by a wide range of physicians. Weaknesses of the study include a limited sample size, which should be addressed in future studies. An additional weakness of the study is the fact that VExUS images were interpreted by blinded attending physicians, rather than by the scanners that acquired the images, which would more closely mirror clinical practice. It was felt that having attending physicians from multiple specialties interpret images would provide a more convincing conclusion than internal medicine residents alone. Furthermore, given that the resident scanners would have additional knowledge about the patient’s degree of congestion (based, e.g., on physical exam characteristics), we sought to avoid the remote risk of unblinding due to characteristics of the ultrasound images that could not be deidentified. Information about the current cohort is also limited; for example, data on CVP was not recorded for these patients. However, VExUS has previously been shown to correlate closely with intracardiac pressures measured by right heart catheterization [9, 10]. Though the interpretation team included a broad range of specialties, the presence of a nephrologist would have made the conclusions more robust. One statistical limitation is that while KS and ICC are standard techniques for assessing IRR and IUR, they measure only the presence, rather than degree of disagreement, meaning that the magnitude of inter-rater disagreement is not assessed. The current study provides the basis for future investigations into effective methodologies for teaching VExUS, as well as the impact of training and experience on IRR and IUR, intra-observer/operator reproducibility, as well as the possibility of change in VExUS score following therapeutic intervention. While more study is needed, these preliminary results suggest that the VExUS score is likely to be a reliable and reproducible measure interpretable by clinicians with diverse backgrounds when paired with an ECG tracing.

## Data Availability

The authors will happily provide data upon request.
